# Treatment patterns and determinants of persistence with advanced therapies in adults with newly diagnosed Crohn’s disease: an interim analysis of an inception cohort registry study (iCREST-CD)

**DOI:** 10.1093/ecco-jcc/jjag167

**Published:** 2026-11-07

**Authors:** Shinichiro Shinzaki, Katsuyoshi Matsuoka, Toshimitsu Fujii, Ryuichi Okamoto, Akihiro Yamada, Reiko Kunisaki, Minoru Matsuura, Kenji Watanabe, Hisashi Shiga, Noritaka Takatsu, Shigeki Bamba, Yohei Mikami, Takayuki Yamamoto, Takahiro Shimoyama, Satoshi Motoya, Takehiro Torisu, Taku Kobayashi, Naoki Ohmiya, Masayuki Saruta, Koichiro Matsuda, Takayuki Matsumoto, Hiroshi Nakase, Atsuo Maemoto, Shinichi Yoshigoe, Hiroaki Tsuchiya, Akiko Hori-Nishi, Tadakazu Hisamatsu, iCREST-CD Study Group, Masakazu Nagahori, Daisuke Saito, Mikio Kawai, Atsushi Masamune, Mitsuo Nagasaka, Tomoe Kazama

**Affiliations:** Department of Gastroenterology, Hyogo Medical University School of Medicine, Nishinomiya, Hyogo, 663-8501, Japan; Division of Gastroenterology and Hepatology, Department of Internal Medicine, Toho University Sakura Medical Centre, Sakura, Chiba, 285-8741, Japan; Department of Gastroenterology and Hepatology, Institute of Science Tokyo, Bunkyo-ku, Tokyo, 113-8519, Japan; Department of Gastroenterology and Hepatology, Institute of Science Tokyo, Bunkyo-ku, Tokyo, 113-8519, Japan; Division of Gastroenterology and Hepatology, Department of Internal Medicine, Toho University Sakura Medical Centre, Sakura, Chiba, 285-8741, Japan; Inflammatory Bowel Disease Centre, Yokohama City University Medical Centre, Minami-ku, Yokohama, Kanagawa, 232-0024, Japan; Department of Gastroenterology and Hepatology, Kyorin University School of Medicine, Mitaka, Tokyo, 181-0004, Japan; Department of Internal Medicine for Inflammatory Bowel Disease, Toyama University Hospital, Sugitani, Toyama, 930-0194, Japan; Division of Gastroenterology, Tohoku University Graduate School of Medicine, Sendai, Miyagi, 980-8574, Japan; Department of Inflammatory Bowel Disease Centre, Fukuoka University Chikushi Hospital, Chikushino, Fukuoka, 818-8502, Japan; Division of Digestive Endoscopy, Shiga University of Medical Science, Otsu, Shiga, 520-2192, Japan; Division of Gastroenterology and Hepatology, Department of Internal Medicine, Keio University, School of Medicine, Shinjuku-ku, Tokyo, 160-8582, Japan; Department of Surgery and Inflammatory Bowel Disease Centre, Yokkaichi Hazu Medical Centre, Yokkaichi, Mie, 510-0016, Japan; Department of Surgery and Inflammatory Bowel Disease Centre, Yokkaichi Hazu Medical Centre, Yokkaichi, Mie, 510-0016, Japan; IBD Centre, Hokkaido Preventive Welfare Federation of Agricultural Cooperative, Sapporo-Kosei General Hospital, Sapporo, Hokkaido, 060-0033, Japan; Department of Medicine and Clinical Science, Graduate School of Medical Sciences, Kyushu University, Higashi-ku, Fukuoka, 812-8582, Japan; Centre for Advanced IBD Research and Treatment, Kitasato University Kitasato Institute Hospital, Minato-ku, Tokyo, 108-8642, Japan; Department of Gastroenterology, Fujita Health University School of Medicine, Toyoake, Aichi, 470-1192, Japan; Division of Gastroenterology and Hepatology, Department of Internal Medicine, The Jikei University School of Medicine, Minato-ku, Tokyo, 105-8471, Japan; Department of Gastroenterology, Matsuda Paediatric & Gastroenterology IBD Clinic, Ishikawa, 920-0981, Japan; Division of Gastroenterology, Department of Internal Medicine, School of Medicine, Iwate Medical University, Morioka, Iwate, 020-0021, Japan; Division of Gastroenterology and Hepatology, Department of Internal Medicine, Sapporo Medical University School of Medicine, Sapporo, Hokkaido, 060-8543, Japan; IBD Centre, Sapporo Higashi Tokushukai Hospital, Sapporo, Hokkaido, 065-0033, Japan; Medical Affairs Division, Janssen Pharmaceutical K.K, Chiyoda-ku, Tokyo, 101-0065, Japan; Medical Affairs Division, Janssen Pharmaceutical K.K, Chiyoda-ku, Tokyo, 101-0065, Japan; Medical Affairs Division, Janssen Pharmaceutical K.K, Chiyoda-ku, Tokyo, 101-0065, Japan; Department of Gastroenterology and Hepatology, Kyorin University School of Medicine, Mitaka, Tokyo, 181-0004, Japan

**Keywords:** Crohn’s disease, drug persistence, early initiation

## Abstract

**Background and Aims:**

Multiple advanced therapies are available for the treatment of Crohn’s disease (CD). Treatment patterns in newly diagnosed patients and the factors associated with persistence remain unclear. We present interim results of the iCREST-CD registry describing treatment patterns and persistence with advanced therapies.

**Methods:**

Patients aged ≥16 years with newly diagnosed CD and who had no prior exposure to advanced therapies were enrolled. Persistence of advanced therapies was estimated using Kaplan–Meier analyses, and associated factors were identified using multivariable Cox proportional hazards models. Persistence was also compared between patients who initiated therapy early or later after diagnosis.

**Results:**

Of 672 patients (68.2% male; mean age 29.4 ± 13.1 years), 498 (74.1%) initiated advanced therapy: infliximab (IFX; 121), adalimumab (ADA; 244), ustekinumab (UST; 107), or vedolizumab (VDZ; 26). Advanced therapy was initiated within 1 year of diagnosis in 404 (60.1%). The 36-month treatment persistence rates for IFX, ADA, UST, and VDZ were 77.5%, 75.7%, 79.8%, and 49.0%, respectively. After propensity score adjustment using inverse probability of treatment weighting, the early initiation was associated with greater persistence than delayed initiation, although the difference did not reach statistical significance (hazard ratio, 0.61; 95% confidence interval, 0.35-1.09; *P* = .067).

**Conclusions:**

A high prevalence of early advanced therapy use and a high level of persistence with first-line advanced therapy were identified in this national cohort. Early initiation was associated with greater treatment persistence than delayed initiation, suggesting the potential clinical relevance of earlier advanced therapy use in routine practice. These findings require confirmation in other prospective studies aiming to evaluate early intervention strategies.

**Registration:**

The study was registered with the University Hospital Medical Information Network Clinical Trials Registry (umin.ac.jp/ctr Identifier: UMIN000032237) prior to initiation.

## 1. Introduction

Crohn’s disease (CD) is an idiopathic, chronic inflammatory disorder of the gastrointestinal (GI) tract that arises from a complex interplay between genetic susceptibility, the immune response, environmental factors, and the gut microbiota.^1-3^ CD is characterized by discontinuous areas of transmural inflammation throughout the GI tract, most commonly affecting the terminal ileum, colon, and perianal region.^4^

The global prevalence of CD has increased markedly, particularly in developed countries, which suggests an important role for environmental factors and early-life exposures in the pathogenesis of the disease.^5^^,^^6^ In Japan, once considered to be a low-risk location,^7^ the number of patients with CD has surged, with the prevalence increasing from 0.88 per 100 000 in 1965 to 77.0 per 100 000 in 2023.^8^^,^^9^

CD typically affects individuals in their second to fourth decades of life and is characterized by recurrent symptoms that significantly impair daily functioning.^10^ At the time of diagnosis of CD, most patients show an inflammatory disease phenotype, but approximately half subsequently develop complications such as strictures, fistulas, or abscesses, which often require surgical interventions.^11^

Current treatment strategies for CD aim not only to control the symptoms, but also to modify the disease course and prevent irreversible bowel damage.^10^ The early use of advanced therapies has been proposed on the basis that treatment initiated earlier in the disease course may improve long-term outcomes.^12^^,^^13^ However, in many previous studies, biologic therapies were initiated at a range of time points following the diagnosis, and the effects of early initiation in routine clinical practice have not been well characterized.^14^^,^^15^ More recent clinical studies have demonstrated the potential benefit of the early introduction of advanced therapies, although the relevance of these findings to real-world clinical settings remains to be determined.^16^^,^^17^ Therefore, real-world evidence regarding the timing of initiation of advanced therapies and its relationship with persistence with treatment is needed to inform clinical decision-making.

In Japan, infliximab (IFX) was the first advanced therapy that was approved for the treatment of CD, in 2002, followed by adalimumab (ADA) in 2010, ustekinumab (UST) in 2017, and vedolizumab (VDZ) in 2019. More recently, additional advanced agents have become available, including risankizumab, mirikizumab, guselkumab, and the Janus kinase inhibitor upadacitinib. Under the Japanese national health insurance system, the prior use of corticosteroids or immunomodulators is not mandatory before the initiation of advanced therapy. With the expanding availability of advanced therapies for CD, including biologics and small molecules, increasing attention has been paid to the timing of their introduction. The early initiation of advanced therapy has been proposed as a strategy to improve the long-term outcomes of patients by targeting disease activity before irreversible bowel damage occurs. Therefore, robust real-world evidence regarding the timing of initiation of advanced therapies and its relationship with treatment persistence is needed to inform clinical decision-making and optimize long-term disease management.

Here, we present an interim analysis (cutoff date: June 30, 2022) of data from the iCREST-CD, a prospective observational registry study conducted in Japan in which patients with newly diagnosed CD were enrolled, to identify real-world treatment patterns, persistence with the use of advanced therapies, and the factors associated with this persistence in patients with CD.

## 2. Materials and methods

### 2.1. Study design

iCREST-CD is a prospective, non-interventional, longitudinal observational registry study being conducted at 19 tertiary centers across Japan. The overall study design has been described elsewhere.^18^ Briefly, patients who were newly diagnosed with CD on or after June 1, 2016 and had not previously been exposed to advanced therapies before their diagnosis of CD were enrolled. The present study was an interim analysis of data in the iCREST-CD cohort registry collected up to June 30, 2022 (the cutoff date for the data used in the interim analysis). The median duration of the observation period for the participants was 44.5 months (range: 0.7-73.0 months) for the 672 enrolled patients.

The study was conducted in accordance with the Ethics Guidelines for Medical and Health Research Involving Human Subjects (December 22, 2014; Ministry of Education, Culture, Sports, Science, and Technology, and Ministry of Health, Labor, and Welfare; partially revised on February 28, 2017) and the World Medical Association Declaration of Helsinki. The study protocol was approved by the ethics committee or institutional review board of each of the participating institutions, and written informed consent was obtained from all the participants.

### 2.2. Patient sample

Male and female patients aged ≥16 years who were newly diagnosed with CD according to the diagnostic criteria of the Ministry of Health, Labor, and Welfare of Japan were enrolled. The timing of the diagnosis of CD was defined using the calendar date on which CD was diagnosed. The baseline (“at diagnosis”) data were defined as the measurement(s) made closest to the date of diagnosis (±3 months), before the initiation of treatment.

Patients were identified by the treating physicians using their medical records, and relevant clinical data were collected. Treatment decisions were made at the discretion of the attending physician. The treatments administered included corticosteroids (prednisolone, methylprednisolone, and budesonide), immunomodulators (azathioprine and mercaptopurine hydrate), and advanced therapies (IFX, ADA, UST, and VDZ). Patients were enrolled consecutively during routine clinical visits to minimize selection bias and were followed for at least 2 years during the observation period.

Patients who had previously used advanced therapies approved for the treatment of autoimmune disease before a diagnosis of CD, those without a clear date of the diagnosis, and those planning to enroll in other interventional or registry studies were excluded.

### 2.3. Data collection

During the observation period, data were collected for all the patients, including their baseline demographics and disease characteristics and the changes in these parameters over time. The collected variables included the Harvey–Bradshaw Index (HBI), circulating C-reactive protein (CRP) concentration, and details of their medical treatments. The early initiation of an advanced therapy was defined as initiation ≤3 months after a diagnosis, and delayed initiation was defined as initiation >3 months after a diagnosis. The 3‑month threshold was selected a priori to identify patients who initiated an advanced therapy during the initial management phase following the diagnosis. A sensitivity analysis using a 6-month threshold was also conducted. Details of the disease activity and laboratory data were also collected at the time of initiation of the first advanced therapy, where available.

### 2.4. Assessments made

The assessments made during the study included the assessment of information on the treatments administered from the time of CD diagnosis, the persistence rate associated with the initial advanced therapy, and the factors associated with this persistence. Discontinuation was defined as the permanent cessation of or a change to the initial advanced therapy.

### 2.5. Statistical methods

Data were analyzed for all the patients for whom records regarding the treatment of CD were available. Descriptive statistics are used to summarize the characteristics and treatment status of the patients. Continuous datasets are summarized using the mean and standard deviation (SD) or median and range, and categorical datasets are summarized using frequencies and proportions. Missing data were handled according to the available data for each analysis; patients with missing values for specific variables were excluded from analyses involving these variables.

The longitudinal treatment patterns for first-line treatment with advanced therapies were visualized using Sankey diagrams. The mean change in HBI from baseline (defined as the date of initiation of the first advanced therapy) and the corresponding 95% confidence intervals (CIs) were estimated at each time point to evaluate the effectiveness of the treatment.

A time-to-event outcome was defined for the evaluation of the persistence of treatment: the time period from the initiation of the first advanced therapy to treatment discontinuation. Patients were censored at the time of loss to follow-up or at the cutoff date for data collection. The median time period to the event and the associated 95% CIs were estimated using the Kaplan–Meier method, and persistence rates were visualized using Kaplan–Meier curves. The log-rank test was used to compare the persistence of therapy between patients who initiated an advanced therapy early after the diagnosis or later.

Cox proportional hazards regression models were used to identify factors associated with discontinuation of advanced therapies. Hazard ratios (HRs) and 95% CIs were estimated using univariable and multivariable models. The variables included in the multivariable analysis were selected on the basis of clinical relevance and the results of preliminary univariable analyses, with a stepwise selection approach being applied, owing to the limited sample size. The estimated HRs and 95% CIs were visualized using forest plots.

A propensity score (PS) was calculated for each advanced therapy, to adjust for differences in patient characteristics when the effectiveness of and persistence of treatment using IFX, ADA, and UST were compared. The covariates included in the PS models were the period during which the initial treatment using an advanced therapy was initiated, age of the patient at this time, disease location (L1–L3), disease behavior (B1–B3), HBI severity category, type of patient care, body mass index (BMI), and circulating CRP concentration. For the comparison between patients in whom advanced therapy was initiated early or later, calendar date was included as a covariate in the PS model, given its potential association with both the likelihood of early treatment initiation and persistence with treatment. In contrast, calendar date was not included in the PS models for the drug-to-drug comparisons, because the availability of specific agents strongly overlapped with the calendar date, which could have led to model instability. PSs were estimated using a multinomial logistic regression model that included sex, age at the initiation of the first advanced therapy, BMI, CRP concentration, disease location, disease behavior, the presence of perianal lesions, the severity of HBI, and prior corticosteroid use. The baseline characteristics of the patients before and after PS adjustment were compared using standardized differences, and the results are presented in Table S2.

For analyses of data collected at the time of CD diagnosis, the covariate set also included the period during which a diagnosis was made, the age of the patient at diagnosis, and their sex. PSs were estimated using multinomial logistic regression, and the outcomes (mean HBI and treatment persistence) were analyzed using inverse probability of treatment weighting (IPTW).

All the statistical analyses, except for graphical visualizations, were performed using SAS v.9.4 (SAS Institute Inc., Cary, NC, USA).

## 3. Results

### 3.1. Patient demographics and disease characteristics at the time of diagnosis of CD

Data for a total of 672 eligible patients (68.2% male), with a mean age (±SD) of 29.4 (±13.1) years, were analyzed. Overall, 54.9% (368/670) of the patients were employed, and 5.1% (34/670) had a family history of CD. According to the Montreal classification, the most common disease location was L3 (ileocolonic; 59.4% [389/655]), and the predominant disease behavior was B1 (non-stricturing, non-penetrating; 62.8% [417/664]). Perianal disease was present in 48.8% of the patients (Table 1).

**Table 1.** Patient demographics and disease characteristics at the time of a diagnosis of Crohn’s disease.

Patient characteristic	Category (*N* = 672)	Data
**Age (years), mean (SD) (*n*** = **672)**		29.4 (13.1)
**BMI (kg/m^2^), mean (SD) (*n*** = **580)**		20.51 (3.45)
**Sex, *n* (%), (*n*** = **672)**	Male	458 (68.2)
	Female	214 (31.8)
**Social status, *n* (%), (*n*** = **670)**	Employed	368 (54.9)
	Student	202 (30.1)
	Homemaker	39 (5.8)
	Unemployed	26 (3.9)
	Others	35 (5.2)
**Family history, *n* (%), (*n*** = **670)**	CD	34 (5.1)
	UC	25 (3.7)
**CRP (mg/L), mean (SD)**		28.0 (35.7)
**Disease location, *n* (%), (*n*** = **655)**	L1: Ileal	155 (23.7)
	L2: Colonic	111 (16.9)
	L3: Ileocolonic	389 (59.4)
**Disease behavior, *n* (%), (*n*** = **664)**	B1: Non-stricturing, non-penetrating	417 (62.8)
	B2: Stricturing	171 (25.8)
	B3: Penetrating	72 (10.8)
	Perianal lesion	324 (48.8)

Abbreviations: BMI, body mass index; CD, Crohn’s disease; CRP, C-reactive protein concentration; SD, standard deviation; UC, ulcerative colitis.

### 3.2. Treatment patterns

The cumulative proportions of the patients who were prescribed corticosteroids, immunomodulators, and advanced therapies 12 and 24 months after a diagnosis of CD were 43.6%, 36.0%, and 60.1%, respectively, after 12 months, and 49.0%, 43.6%, and 68.8%, respectively, after 24 months (Figure 1). Advanced therapy was initiated within 1 year of diagnosis in 404 (60.1%) patients. A Sankey diagram illustrates the treatment trajectories of the patients with respect to advanced therapies as far as the third-line therapy (Figure 2).

**Figure 1.**
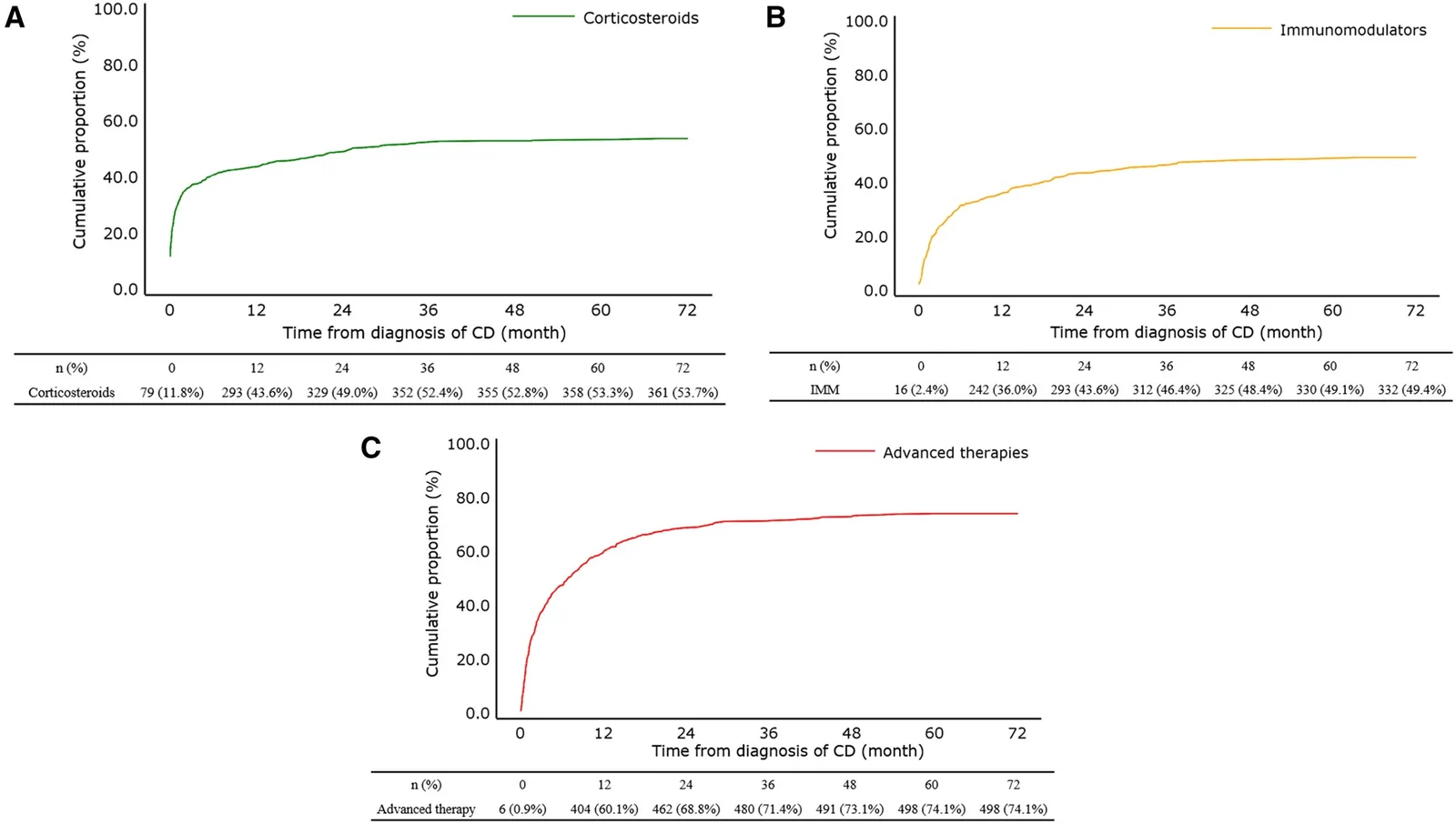
Cumulative proportion of the patients who were prescribed treatments following a diagnosis of CD. (A) Corticosteroids, (B) immunomodulators, (C) advanced therapy. Abbreviations: CD, Crohn’s disease; IMM, immunomodulators.

**Figure 2.**
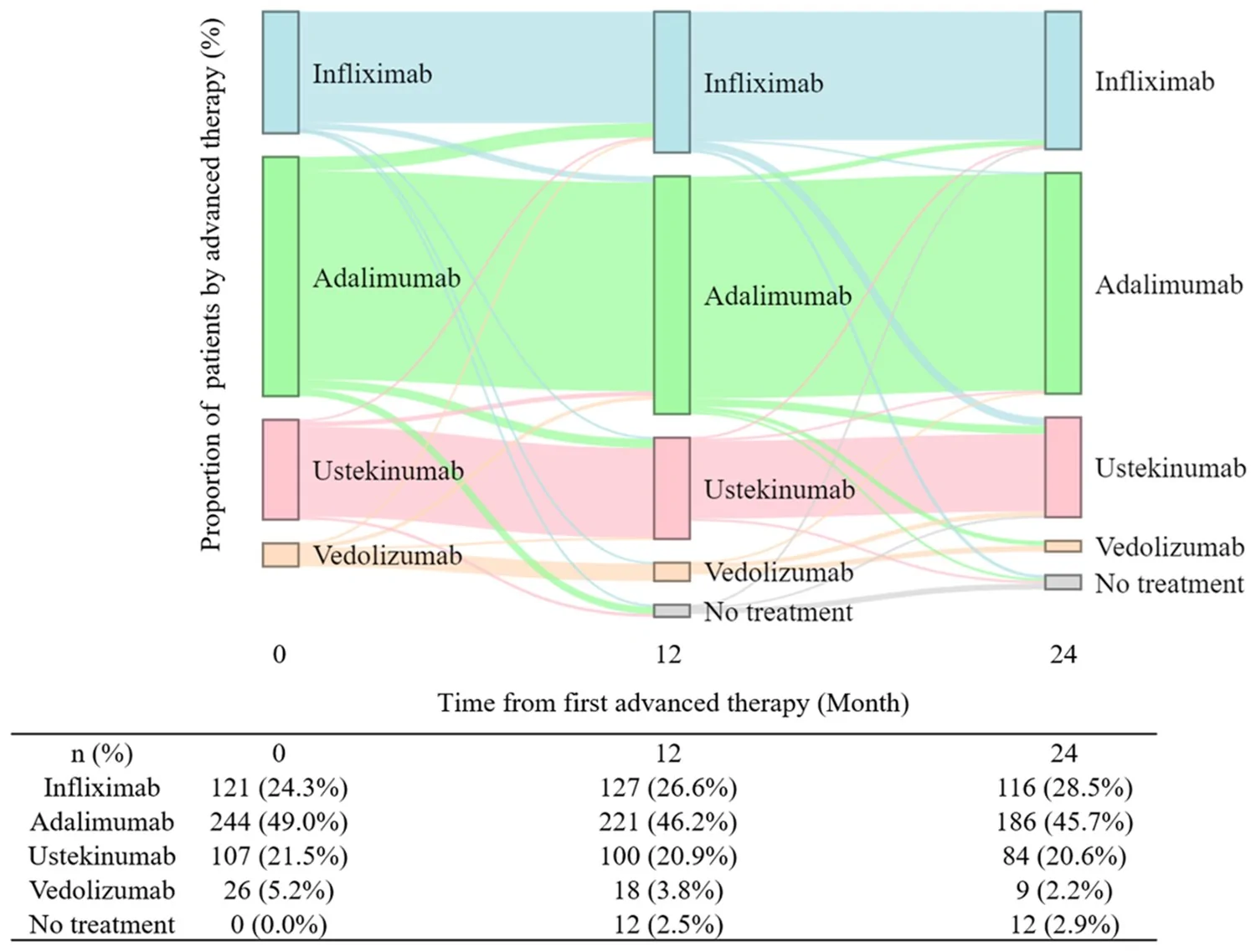
Sankey diagram illustrating the treatment trajectories of the patients from the initiation of their first-line advanced therapy.

### 3.3. Patient demographics and disease characteristics, according to the advanced therapy administered

A total of 498 (74.1%) patients initiated treatment with the following advanced therapies: IFX, 121; ADA, 244; UST, 107; and VDZ, 26 (Table 2). At the time of initiation of their first-line advanced therapy with IFX, ADA, UST, or VDZ, the mean ages of the patients were 28.2, 26.6, 30.4, and 36.2 years, respectively. The median interval from a diagnosis of CD to the initiation of advanced therapy was 1.4 months (range, 0.0-48.0 months) for IFX, 2.8 months (range, 0.0-59.6 months) for ADA, 5.5 months (range, 0.0-54.8 months) for UST, and 11.7 months (0.4-52.1 months) for VDZ (Table 2). The mean CRP concentrations of the patients at the initiation of IFX, ADA, UST, and VDZ were 25.9, 18.9, 13.5, and 9.5 mg/L, respectively (Table 2). After PS adjustment using IPTW, the baseline characteristics of the treatment groups were similar, with low standardized differences (Table S2).

**Table 2.** Patient demographics and disease characteristics at the time of initiation of the first-line advanced therapy.

Total *n* = 672		First-line advanced therapy, *n* = 498 (74.1%)			
		Infliximab	Adalimumab	Ustekinumab	Vedolizumab
		*n* (%)	*n* (%)	*n* (%)	*n* (%)
**Number of patients**	*n* (%)	121 (18.0%)	244 (36.3%)	107 (15.9%)	26 (3.9%)
**Age (years)**	*n*	121	244	107	26
	Mean ± SD	28.2 ± 11.3	26.6 ± 10.8	30.4 ± 14.1	36.2 ± 17.4
**Time from a diagnosis of CD to the initiation of an advanced therapy (months)**	*n*	121	244	107	26
	Median (Min, Max)	1.4 (0.0, 48.0)	2.8 (0.0, 59.6)	5.5 (0.0, 54.8)	11.7 (0.4, 52.1)
**Sex**	*n*	121	244	107	26
	Male	87 (71.9%)	167 (68.4%)	66 (61.7%)	16 (61.5%)
	Female	34 (28.1%)	77 (31.6%)	41 (38.3%)	10 (38.5%)
**Duration of follow-up (months)**	*n*	121	244	107	26
	Median (Min, Max)	44.6 (1.1, 71.9)	41.1 (3.5, 72.2)	28.9 (1.9, 59.7)	23.1 (8.8, 36.6)
**BMI (kg/m^2^)**	*n*	108	218	94	21
	Mean ± SD	20.25 ± 3.69	20.45 ± 3.09	20.47 ± 3.42	22.26 ± 4.54
**Disease location**	*n*	105	219	102	23
	L1: Ileal	24 (22.9%)	50 (22.8%)	40 (39.2%)	6 (26.1%)
	L2: Colonic	22 (21.0%)	35 (16.0%)	8 (7.8%)	7 (30.4%)
	L3: Ileocolonic	59 (56.2%)	134 (61.2%)	54 (52.9%)	10 (43.5%)
**Disease behavior**	*n*	117	240	107	26
	B1: Non-stricturing, non-penetrating	69 (59.0%)	141 (58.8%)	62 (57.9%)	11 (42.3%)
	B2: Stricturing	30 (25.6%)	64 (26.7%)	34 (31.8%)	9 (34.6%)
	B3: Penetrating	18 (15.4%)	34 (14.2%)	11 (10.3%)	6 (23.1%)
	Perianal lesion	47 (40.2%)	113 (47.1%)	41 (38.3%)	8 (30.8%)
**CRP (mg/L)**	*n*	117	238	107	26
	Mean ± SD	25.9 ± 26.8	18.9 ± 26.0	13.5 ± 17.9	9.5 ± 19.0
**HBI**	*n*	35	117	60	20
	Mean ± SD	6.1 ± 5.1	5.0 ± 3.7	4.5 ± 4.2	3.2 ± 2.7
**Severity**	*n*	35	117	60	20
	Remission (HBI <5)	15 (42.9%)	60 (51.3%)	34 (56.7%)	13 (65.0%)
	Mild activity (HBI 5-7)	9 (25.7%)	32 (27.4%)	17 (28.3%)	6 (30.0%)
	Moderate activity (HBI 8-16)	10 (28.6%)	24 (20.5%)	8 (13.3%)	1 (5.0%)
	Severe activity (HBI >16)	1 (2.9%)	1 (0.9%)	1 (1.7%)	0 (0.0%)

Abbreviations: BMI, body mass index; CD, Crohn’s disease; min, minimum; max, maximum; CRP, C-reactive protein concentration; HBI, Harvey–Bradshaw index, duration of follow-up, number of months from the initiation of the first-line advanced therapy to the cutoff date for data collection; SD, standard deviation.

### 3.4. Persistence of advanced therapy

The persistence rates for advanced therapies 24 months after initiation were 85.7% for IFX, 79.8% for ADA, 82.0% for UST, and 49.0% for VDZ; and the 36-month persistence rates were 77.5% for IFX, 75.7% for ADA, 79.8% for UST, and 49.0% for VDZ (Figure 3A). Using the IPTW method, the 24-month persistence rates were 88.3% for IFX, 81.6% for ADA, and 78.8% for UST; and the 36-month persistence rates were 80.7% for IFX, 76.6% for ADA, and 77.2% for UST (Figure 3B).

**Figure 3.**
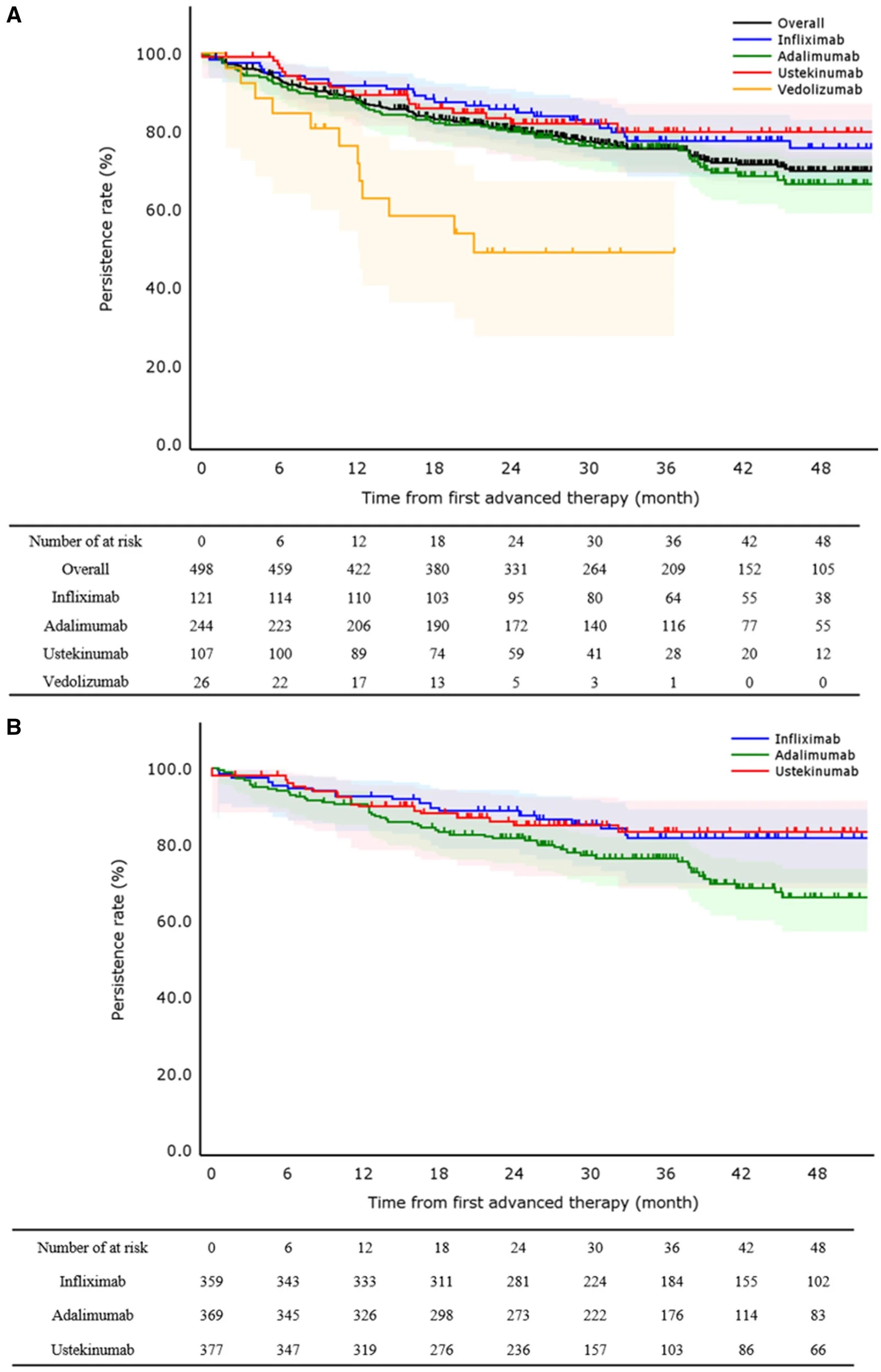
Persistence with administration of the first-line advanced therapy. (A) Results of the unadjusted Kaplan–Meier analysis. (B) Results of the inverse probability of treatment weighting-adjusted Kaplan–Meier analysis.

### 3.5. Factors associated with persistence with advanced therapies

In the multivariable Cox proportional hazards analysis of the use of all the advanced therapies together, male sex (HR, 0.59; 95% CI 0.38-0.93) and prior intestinal resection surgery (HR, 0.19; 95% CI, 0.05-0.83) were associated with a lower risk of discontinuation of the first advanced therapy (Table 3; Figure S1). Disease location L1 was associated with a lower risk of discontinuation than L2 (HR, 0.27; 95% CI, 0.14-0.53), whereas the association for L3 did not reach statistical significance (HR, 0.68; 95% CI, 0.43-1.07) (Table 3; Figure S1). This association remained significant when IFX and ADA were analyzed separately in multivariable models (Table S1).

**Table 3.** Results of the Cox proportional hazards analysis to identify factors associated with the discontinuation of the first-line advanced therapy.

				Hazard ratio^a^			Adjusted hazard ratio^b^		
Risk factor	Category	*N*	Event	HR	Lower 95% CI	Upper 95% CI	HR	Lower 95% CI	Upper 95% CI
**Age at the initiation of the first advanced therapy (years)**		498	126	1.01	0.99	1.02	1.01	1.00	1.03
**Sex**	Female	162	51	Reference			Reference		
	Male	336	75	0.66	0.46	0.94	0.59	0.38	0.93
**Corticosteroid use before the first advanced therapy**	No	377	93	Reference			Reference		
	Yes	121	33	1.23	0.83	1.84	0.80	0.51	1.26
**Immunomodulator use before the first advanced therapy**	No	379	100	Reference			Reference		
	Yes	119	26	0.81	0.53	1.25	0.76	0.48	1.21
**Early or delayed initiation of an advanced therapy**	Delayed initiation	245	63	Reference			Reference		
	Early initiation	253	63	0.85	0.60	1.21	0.72	0.47	1.09
**Intestinal resection surgery before the first advanced therapy**	No	455	124	Reference			Reference		
	Yes	43	2	0.16	0.04	0.66	0.19	0.05	0.83
**Disease location**	L2: Colonic	79	31	Reference			Reference		
	L1: Ileal	130	15	0.26	0.14	0.48	0.27	0.14	0.53
	L3: Ileocolonic	277	77	0.66	0.43	1.00	0.68	0.43	1.07
**Disease behavior**	B1: Non-stricturing, non-penetrating disease	283	82	Reference			Reference		
	B2: Stricturing disease	137	29	0.69	0.45	1.06	1.01	0.64	1.61
	B3: Penetrating disease	69	12	0.58	0.32	1.06	1.00	0.52	1.90
**Perianal lesion**	No	281	69	Reference			Reference		
	Yes	209	55	1.12	0.78	1.59	0.96	0.66	1.40
**Hemoglobin concentration**	≥12.5 g/dL	257	61	Reference			Reference		
	<10 g/dL	52	17	1.40	0.82	2.40	0.69	0.34	1.40
	10-12.49 g/dL	181	46	1.03	0.70	1.51	0.67	0.41	1.09
**Serum albumin concentration**	≥4 g/dL	171	34	Reference			Reference		
	<3 g/dL	63	23	1.92	1.13	3.26	1.72	0.84	3.54
	3-3.99 g/dL	252	67	1.36	0.90	2.05	1.29	0.80	2.09
**C-reactive protein concentration**	3 mg/L	488	124	1.03	1.01	1.04	1.02	1.00	1.04
**Severity**	Remission (HBI <5)	122	30	Reference			Reference		
	Mild activity (HBI 5-7)	64	21	1.30	0.75	2.28	1.02	0.57	1.84
	Moderate activity (HBI 8-16)	43	12	1.06	0.54	2.06	0.74	0.36	1.53
	Severe activity (HBI >16)	3	1	0.88	0.12	6.47	0.45	0.06	3.46

a Hazard ratio for each risk factor, from the Cox proportional hazards model.

b Hazard ratio for each risk factor, from the multivariate Cox proportional hazards model.

Abbreviations: CI, confidence interval; HBI, Harvey–Bradshaw index; HR, hazard ratio.

### 3.6. Persistence with the first advanced therapy, according to the timing of initiation

In unadjusted Kaplan–Meier analyses, no marked difference in persistence with the medication was identified between the early and delayed advanced therapy initiation groups **(**Figure 4A).

**Figure 4.**
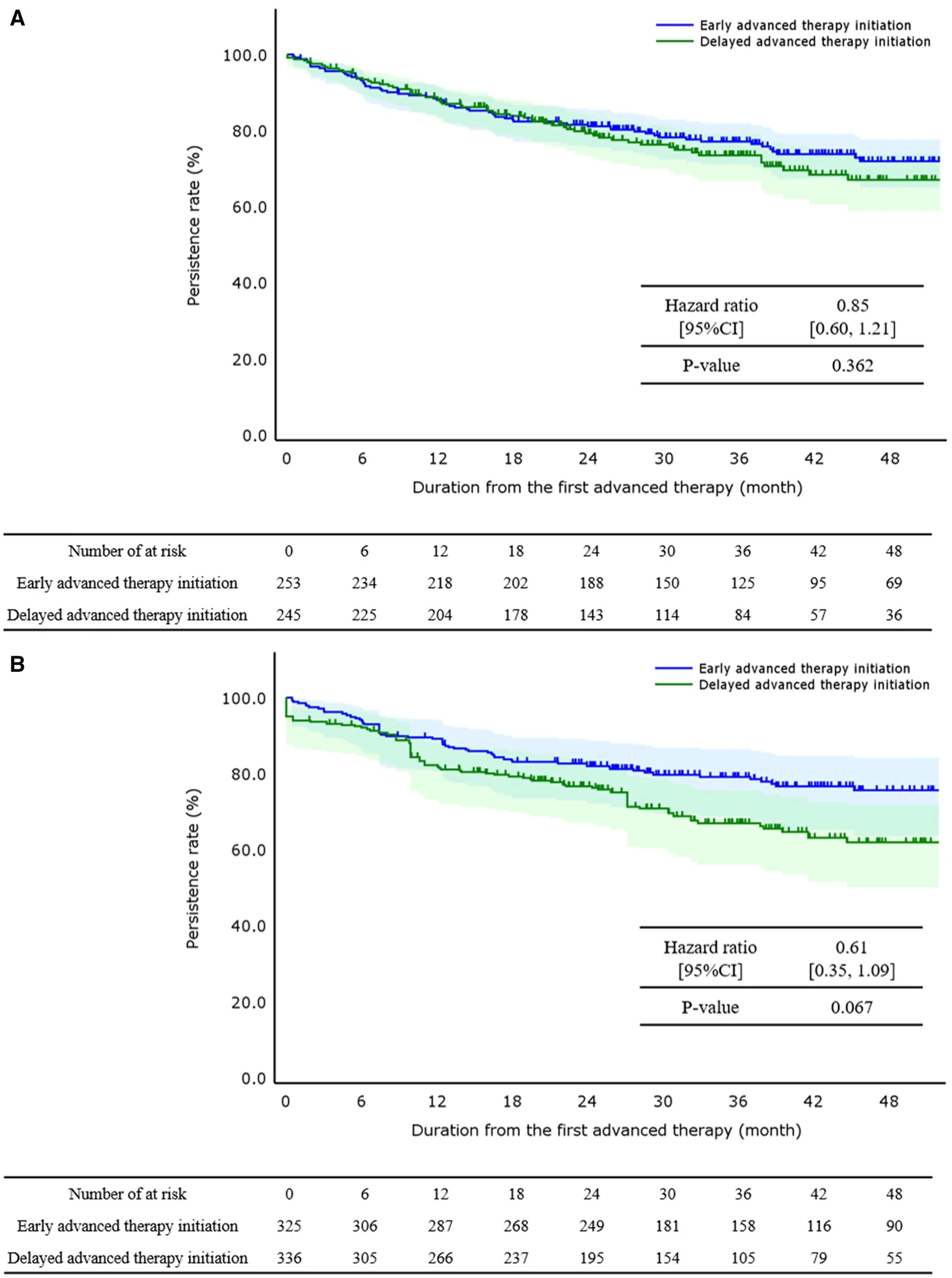
Persistence with the first-line advanced therapy, according to the timing of initiation. (A) Results of the unadjusted Kaplan–Meier analysis. (B) Results of the Kaplan–Meier analysis adjusted using inverse probability of treatment weighting based on propensity scores.

After adjustment using IPTW based on PSs, greater persistence with the first advanced therapy was identified in the early initiation group than in the delayed initiation group up to 48 weeks (Figure 4B). The HR for treatment discontinuation was 0.61 (95% CI, 0.35-1.09), with a log-rank *P*-value of .067, suggesting a trend toward greater persistence following early initiation, albeit the difference was not statistically significant.

As a sensitivity analysis, persistence was also evaluated using a 6-month threshold to differentiate early and delayed initiation; the unadjusted Kaplan–Meier curves are shown in Figure S2.

## 4. Discussion

The interim analysis of the iCREST-CD inception cohort discussed herein has provided a contemporary and comprehensive overview of the real-world treatment patterns and outcomes of patients with newly diagnosed CD in Japan. Several key findings were made. First, advanced therapies were introduced early in the disease course in a substantial proportion of the patients, with approximately 60% initiating such therapy within the first year of diagnosis. Consistent with this finding, the cumulative exposure to advanced therapies from the time of diagnosis was greater than that to corticosteroids or immunomodulators (Figure 1). Second, the overall persistence rates with the first-line advanced therapy were high for all the agents, and particularly for anti-tumor necrosis factor therapies and UST. Third, when treatment strategies were evaluated according to the timing of the initiation of the advanced therapies, earlier initiation was associated with greater persistence with the first-line advanced therapy than delayed initiation after adjustment for clinical and demographic parameters.

The present findings differ from those of global real‑world cohort studies, such as the ICURE^14^ and ECCO-EpiCom^15^ studies, which showed greater corticosteroid use and less use of advanced therapies. In the present study, the cumulative proportion of patients using corticosteroids was lower (53.7% [iCREST] vs 76.4% [ICURE]/61%-67% [ECCO-EpiCom]), whereas that for advanced therapies was higher (74.1% [iCREST] vs 20.3% [ICURE]/9%-19% [ECCO-EpiCom]). These differences can be attributed to factors such as the time periods during which the studies were conducted (ICURE is an older study, conducted when fewer advanced therapies were available), regional differences (Asian vs European/Western settings), the adoption of the Selecting Therapeutic Targets in Inflammatory Bowel Disease (STRIDE) treat‑to‑target initiatives in Japan, the sample size analyzed, and differences in the associated healthcare systems. Importantly, we cannot infer causal links of intractable disease status or the use of subsidies with the use of advanced therapies on the basis of the present data.

With respect to the use of individual advanced therapies, the ECCO-EpiCom study,^15^ VERNE, a retrospective observation study,^19^ and the present study have shown that ADA is the most highly prescribed advanced therapy, followed by IFX. IFX treatment was often initiated for patients with a high HBI and high CRP concentration early after a diagnosis was made. The lower persistence identified for VDZ should be interpreted with caution because the VDZ group was small and may have differed from other treatment groups with respect to baseline characteristics, the timing of drug availability, and treatment selection by the physicians.

In the present study, patients with ileocolonic involvement (L3), penetrating behavior (B3), or clinical activity of their disease at the time of diagnosis were more likely to commence an advanced therapy early. In multivariable Cox regression analyses, ileal disease (L1) was associated with greater treatment persistence than colonic disease (L2), which may reflect differences in disease severity or symptom burden, but this finding should be interpreted with caution.

The conventional management of CD used to involve a step‑up approach, with escalation to the use of advanced therapies after the failure of conventional therapy. However, recent guidelines endorse the earlier use of advanced therapies, particularly for patients who are at risk of disease progression.^12^^,^^13^ Compared with conventional step‑up management, the early use of combined immunosuppression yielded higher remission rates and faster improvements in the CD Activity Index and CRP concentration in a randomized trial by D’Haens et al.^16^ More recently, the PROFILE trial provided compelling evidence that a biomarker‑stratified early advanced therapy strategy achieved superior steroid‑ and surgery‑free remission than accelerated step‑up therapy in patients with newly diagnosed CD.^17^ Nevertheless, PROFILE was designed as a precision‑medicine trial, in which patients were enrolled on the basis of predefined prognostic biomarkers and protocol‑driven treatment escalation was used. Although this design meant that the trial had high internal validity, the findings were inherently limited regarding the insight gained into how successful early treatment with advanced therapies can be in patients managed at the discretion of the physician, according to their own preference, and according to the constraints of the healthcare system.^17^ In contrast, the iCREST‑CD registry captures real-world treatment decisions made at the discretion of the physician, without mandated escalation rules, biomarker preselection, or protocol‑enforced treatment changes.^18^ Within this practical setting, the earlier initiation of advanced therapy was associated with greater persistence with the first-line treatment, irrespective of the class of agent used, after adjustment for clinical characteristics. It should be noted that the definition of early treatment varies between studies. In many clinical settings, advanced therapies are initiated some time after the onset of symptoms, even if their initiation is classified as “early,” according to the period of time elapsed since the diagnosis. Because in the present study we defined early initiation according to the period of time elapsed since the diagnosis, some of the patients may have had their disease for a long period prior to diagnosis. This limitation should be considered when interpreting the findings.

These findings indicate that the benefits of early advanced therapy use demonstrated in controlled trials may extend to routine clinical practice. Moreover, whereas randomized trials such as that by D’Haens et al.^16^ and PROFILE^17^ focused primarily on remission endpoints at fixed timepoints, the present study demonstrates that persistence with treatment is a clinically meaningful and complementary outcome. Although long-term persistence with the first-line advanced therapy is not a direct measure of efficacy, it represents a clinically meaningful outcome that reflects a composite of sustained effectiveness, tolerability, and patient acceptance, which are critical determinants of real-world success, but are not fully captured by the short-term remission rate. However, persistence may also be influenced by non-clinical factors, and should not be interpreted as a surrogate for efficacy alone. The identified trend toward greater persistence following the early initiation of an advanced therapy was supported by both the IPTW-adjusted analysis (Figure 4B) and the multivariable Cox proportional hazards model (Table 3), albeit that the finding was not statistically significant. Because this analysis was based on observational registry data, the findings should be considered to be hypothesis-generating and require confirmation in studies of independent prospective cohorts aiming to evaluate early intervention strategies.

It should also be noted that the concept of early intervention has evolved alongside advances in therapeutic options and treatment paradigms. As a result, the date of treatment is intrinsically linked to the likelihood of the early initiation of an advanced therapy. Therefore, although adjustment for calendar date is necessary to reduce confounding in observational analyses, such an adjustment may account for aspects of the treatment strategy itself and attenuate the strength of the identified relationship. Accordingly, the present findings should be interpreted as reflecting the association between the early initiation of an advanced therapy and persistence with the treatment within the context of the evolution of clinical practice.

One of the limitations of the present study was that the data were collected primarily from centers specializing in CD, and therefore they may not be representative of the entire population of Japan. In addition, registry-based real-world studies have several inherent limitations, including missing data, a lack of follow-up, and inconsistent data collection. In addition, the fecal calprotectin content, the concentrations of biologic agents, anti-drug antibody titers, ethnicity, and cost-effectiveness were not systematically assessed, and the generalizability of the findings to older and pediatric patients requires further evaluation. Furthermore, no endoscopic assessments were included in the present study. Data collected regarding the use of advanced therapies, including risankizumab, mirikizumab, guselkumab, and upadacitinib, which were approved after the enrollment period, were not assessed in the present study.

In conclusion, in this large real-world registry study, we have demonstrated that treatment with advanced therapies was frequently initiated early after the diagnosis of CD in Japan. The early initiation of an advanced therapy was associated with greater persistence with the treatment than delayed initiation, highlighting the potential clinical importance of the timing of treatment for long-term disease management. These findings provide real-world evidence that complements the results of controlled trials and suggest that early treatment with an advanced therapy may be associated with greater treatment persistence in appropriate patients. However, these findings should be confirmed in studies of other prospective cohorts and early intervention strategies.

## Supplementary Material

jjag167_Supplementary_Data

## Data Availability

Not applicable. The data used in this study were obtained under a confidentiality agreement with Janssen Pharmaceutical K.K. and cannot be shared publicly.

## Appendix

Collaborators

iCREST-CD Study Group:

Masakazu Nagahori, Health Science Research & Development Center, Institute of Science Tokyo; Daisuke Saito, Department of Gastroenterology and Hepatology, Kyorin University School of Medicine; Mikio Kawai, Department of Internal Medicine, Hyogo Medical University; Atsushi Masamune, Division of Gastroenterology, Tohoku University Graduate School of Medicine; Mitsuo Nagasaka, Department of Gastroenterology, Fujita Health University School of Medicine; Tomoe Kazama, Department of Gastroenterology and Hepatology, Sapporo Medical University School of Medicine.
