# Supplementary material for: Treatment patterns and determinants of persistence with advanced therapies in adults with newly diagnosed Crohn’s disease: an interim analysis of an inception cohort registry study (iCREST-CD)
Source: J Crohns Colitis. 2026 Nov 7;20(11):jjag167. doi: 10.1093/ecco-jcc/jjag167 (PMC13744011; doi:10.1093/ecco-jcc/jjag167)
Supplement: Supplementary file 1 [file jjag167_supplementary_data.zip › Shinzaki Matsuoka_et_al_iCREST-CD_Supplementary_revision_20260803.docx]

**Supplementary Data**

**Table S1.** **Hazard ratios for the factors associated with the duration of treatment with the first advanced agent selected, obtained using univariate and multivariate Cox models**

|  |  |  |  |  | Hazard ratio^*1^ | | | Adjusted hazard ratio^*2^ | | |
| --- | --- | --- | --- | --- | --- | --- | --- | --- | --- | --- |
| Advanced agent | Risk factor | Category | N | Event | HR | Lower  95% CI | Upper  95% CI | HR | Lower  95% CI | Upper  95% CI |
| IFX | Age at the initiation of the first advanced therapy (years) |  | 121 | 26 | 1.00 | 0.97 | 1.04 | 1.01 | 0.96 | 1.05 |
|  | Sex | Female | 34 | 11 | Reference |  |  | Reference |  |  |
|  |  | Male | 87 | 15 | 0.48 | 0.22 | 1.04 | 0.40 | 0.14 | 1.20 |
|  | Corticosteroid use before the first advanced therapy | No | 86 | 15 | Reference |  |  | Reference |  |  |
|  |  | Yes | 35 | 11 | 1.97 | 0.90 | 4.29 | 1.25 | 0.44 | 3.54 |
|  | Immunomodulator use before the first advanced therapy | No | 86 | 24 | Reference |  |  | Reference |  |  |
|  |  | Yes | 35 | 2 | 0.18 | 0.04 | 0.74 | 0.10 | 0.02 | 0.55 |
|  | Early/delayed initiation of an advanced therapy | Delayed initiation | 38 | 7 | Reference |  |  | Reference |  |  |
|  |  | Early initiation | 83 | 19 | 1.30 | 0.55 | 3.10 | 0.93 | 0.26 | 3.26 |
|  | Intestinal resection surgery before the first advanced therapy | No | 116 | 26 | Reference |  |  | Reference |  |  |
|  |  | Yes | 5 | 0 | 0.00 | 0.00 | - | 0.00 | 0.00 | - |
|  | Disease location | L2: Colonic | 25 | 10 | Reference |  |  | Reference |  |  |
|  |  | L1: Ileal | 24 | 0 | 0.00 | 0.00 | - | 0.00 | 0.00 | - |
|  |  | L3: Ileocolonic | 66 | 15 | 0.53 | 0.24 | 1.18 | 0.60 | 0.20 | 1.84 |
|  | Disease behaviour | B1: Non-stricturing, non-penetrating disease | 69 | 18 | Reference |  |  | Reference |  |  |
|  |  | B2: Stricturing disease | 30 | 5 | 0.61 | 0.23 | 1.64 | 1.25 | 0.31 | 5.07 |
|  |  | B3: Penetrating disease | 18 | 2 | 0.39 | 0.09 | 1.67 | 0.51 | 0.07 | 4.02 |
|  | Perianal lesions | No | 70 | 16 | Reference |  |  | Reference |  |  |
|  |  | Yes | 47 | 9 | 0.92 | 0.40 | 2.08 | 0.79 | 0.28 | 2.19 |
|  | Haemoglobin concentration | ≥12.5 g/dL | 50 | 9 | Reference |  |  | Reference |  |  |
|  |  | <10 g/dL | 22 | 7 | 1.96 | 0.73 | 5.28 | 0.60 | 0.15 | 2.38 |
|  |  | 10–12.49 g/dL | 45 | 10 | 1.26 | 0.51 | 3.09 | 0.37 | 0.09 | 1.45 |
|  | Serum albumin concentration | ≥4 g/dL | 27 | 2 | Reference |  |  | Reference |  |  |
|  |  | <3 g/dL | 27 | 9 | 5.21 | 1.13 | 24.11 | 4.58 | 0.49 | 43.16 |
|  |  | 3–3.99 g/dL | 61 | 15 | 3.55 | 0.81 | 15.54 | 2.70 | 0.51 | 14.20 |
|  | C-reactive protein concentration | 3 mg/L | 117 | 26 | 1.05 | 1.01 | 1.09 | 1.06 | 1.00 | 1.11 |
|  | Severity | Remission (HBI <5) | 15 | 3 | Reference |  |  | Reference |  |  |
|  |  | Mild activity (HBI 5–7) | 9 | 1 | 0.54 | 0.06 | 5.15 | 0.09 | 0.01 | 1.52 |
|  |  | Moderate activity (HBI 8–16) | 10 | 3 | 1.52 | 0.31 | 7.57 | 0.30 | 0.04 | 2.28 |
|  |  | Severe activity (HBI >16) | 1 | 1 | 4.00 | 0.41 | 38.57 | 0.07 | 0.00 | 3.37 |
| ADA | Age at the initiation of the first advanced therapy (years) |  | 244 | 70 | 1.03 | 1.01 | 1.05 | 1.03 | 1.01 | 1.06 |
|  | Sex | Female | 77 | 25 | Reference |  |  | Reference |  |  |
|  |  | Male | 167 | 45 | 0.78 | 0.48 | 1.28 | 0.80 | 0.43 | 1.50 |
|  | Corticosteroid use before the first advanced therapy | No | 194 | 55 | Reference |  |  | Reference |  |  |
|  |  | Yes | 50 | 15 | 1.19 | 0.67 | 2.12 | 0.70 | 0.33 | 1.50 |
|  | Immunomodulator use before the first advanced therapy | No | 191 | 54 | Reference |  |  | Reference |  |  |
|  |  | Yes | 53 | 16 | 1.07 | 0.61 | 1.88 | 0.94 | 0.51 | 1.76 |
|  | Early/delayed initiation of an advanced therapy | Delayed initiation | 121 | 37 | Reference |  |  | Reference |  |  |
|  |  | Early initiation | 123 | 33 | 0.77 | 0.48 | 1.24 | 0.65 | 0.36 | 1.17 |
|  | Intestinal resection surgery before the first advanced therapy | No | 217 | 70 | Reference |  |  | Reference |  |  |
|  |  | Yes | 27 | 0 | 0.00 | 0.00 | - | 0.00 | 0.00 | - |
|  | Disease location | L2: Colonic | 39 | 17 | Reference |  |  | Reference |  |  |
|  |  | L1: Ileal | 57 | 10 | 0.35 | 0.16 | 0.76 | 0.56 | 0.22 | 1.39 |
|  |  | L3: Ileocolonic | 143 | 41 | 0.58 | 0.33 | 1.01 | 0.98 | 0.50 | 1.94 |
|  | Disease behaviour | B1: Non-stricturing, non-penetrating disease | 141 | 43 | Reference |  |  | Reference |  |  |
|  |  | B2: Stricturing disease | 64 | 18 | 0.81 | 0.47 | 1.41 | 0.96 | 0.50 | 1.82 |
|  |  | B3: Penetrating disease | 34 | 7 | 0.65 | 0.29 | 1.45 | 1.59 | 0.66 | 3.83 |
|  | Perianal lesions | No | 127 | 37 | Reference |  |  | Reference |  |  |
|  |  | Yes | 113 | 32 | 1.04 | 0.65 | 1.68 | 0.93 | 0.55 | 1.57 |
|  | Haemoglobin concentration | ≥12.5 g/dL | 128 | 34 | Reference |  |  | Reference |  |  |
|  |  | <10 g/dL | 19 | 6 | 1.29 | 0.54 | 3.07 | 0.82 | 0.27 | 2.50 |
|  |  | 10–12.49 g/dL | 93 | 28 | 1.11 | 0.68 | 1.84 | 0.84 | 0.44 | 1.60 |
|  | Serum albumin concentration | ≥4 g/dL | 89 | 23 | Reference |  |  | Reference |  |  |
|  |  | <3 g/dL | 28 | 13 | 2.16 | 1.09 | 4.27 | 1.80 | 0.68 | 4.78 |
|  |  | 3–3.99 g/dL | 122 | 32 | 1.04 | 0.61 | 1.77 | 0.94 | 0.49 | 1.78 |
|  | C-reactive protein concentration | 3 mg/L | 238 | 68 | 1.02 | 1.00 | 1.04 | 1.02 | 0.99 | 1.05 |
|  | Severity | Remission (HBI<5) | 60 | 14 | Reference |  |  | Reference |  |  |
|  |  | Mild activity (HBI 5–7) | 32 | 10 | 1.30 | 0.58 | 2.92 | 1.06 | 0.43 | 2.59 |
|  |  | Moderate activity (HBI 8–16) | 24 | 8 | 1.42 | 0.60 | 3.39 | 0.78 | 0.25 | 2.38 |
|  |  | Severe activity (HBI >16) | 1 | 0 | 0.00 | 0.00 | - | 0.00 | 0.00 | - |
| UST | Age at the initiation of the first advanced therapy (years) |  | 107 | 18 | 0.96 | 0.92 | 1.01 | 1.02 | 0.96 | 1.08 |
|  | Sex | Female | 41 | 9 | Reference |  |  | Reference |  |  |
|  |  | Male | 66 | 9 | 0.63 | 0.25 | 1.58 | 0.54 | 0.11 | 2.71 |
|  | Corticosteroid use before the first advanced therapy | No | 76 | 14 | Reference |  |  | Reference |  |  |
|  |  | Yes | 31 | 4 | 0.72 | 0.24 | 2.17 | 1.32 | 0.36 | 4.84 |
|  | Immunomodulator use before the first advanced therapy | No | 85 | 13 | Reference |  |  | Reference |  |  |
|  |  | Yes | 22 | 5 | 1.80 | 0.64 | 5.07 | 1.64 | 0.37 | 7.26 |
|  | Early/delayed initiation of an advanced therapy | Delayed initiation | 65 | 11 | Reference |  |  | Reference |  |  |
|  |  | Early initiation | 42 | 7 | 0.79 | 0.31 | 2.06 | 0.92 | 0.22 | 3.78 |
|  | Intestinal resection surgery before the first advanced therapy | No | 99 | 17 | Reference |  |  | Reference |  |  |
|  |  | Yes | 8 | 1 | 0.95 | 0.13 | 7.17 | 1.26 | 0.12 | 13.10 |
|  | Disease location | L2: Colonic | 8 | 1 | Reference |  |  | Reference |  |  |
|  |  | L1: Ileal | 42 | 3 | 0.53 | 0.06 | 5.10 | 0.17 | 0.01 | 2.71 |
|  |  | L3: Ileocolonic | 57 | 14 | 2.14 | 0.28 | 16.28 | 0.83 | 0.07 | 9.96 |
|  | Disease behaviour | B1: Non-stricturing, non-penetrating disease | 62 | 12 | Reference |  |  | Reference |  |  |
|  |  | B2: Stricturing disease | 34 | 4 | 0.63 | 0.20 | 1.95 | 1.30 | 0.31 | 5.57 |
|  |  | B3: Penetrating disease | 11 | 2 | 1.06 | 0.24 | 4.75 | 7.73 | 0.90 | 66.54 |
|  | Perianal lesion | No | 66 | 7 | Reference |  |  | Reference |  |  |
|  |  | Yes | 41 | 11 | 2.65 | 1.03 | 6.84 | 5.90 | 1.43 | 24.35 |
|  | Haemoglobin concentration | ≥12.5 g/dL | 61 | 9 | Reference |  |  | Reference |  |  |
|  |  | <10 g/dL | 10 | 3 | 1.93 | 0.52 | 7.13 | 1.36 | 0.15 | 12.13 |
|  |  | 10–12.49 g/dL | 36 | 6 | 1.05 | 0.37 | 2.96 | 0.45 | 0.06 | 3.28 |
|  | Serum albumin concentration | ≥4 g/dL | 39 | 6 | Reference |  |  | Reference |  |  |
|  |  | <3 g/dL | 8 | 1 | 0.67 | 0.08 | 5.58 | 0.31 | 0.02 | 4.97 |
|  |  | 3–3.99 g/dL | 59 | 11 | 1.09 | 0.40 | 2.94 | 0.70 | 0.18 | 2.74 |
|  | C-reactive protein concentration | 3 mg/L | 107 | 18 | 1.05 | 0.98 | 1.12 | 1.03 | 0.93 | 1.15 |
|  | Severity | Remission (HBI<5) | 34 | 9 | Reference |  |  | Reference |  |  |
|  |  | Mild activity (HBI 5–7) | 17 | 6 | 1.18 | 0.42 | 3.32 | 1.51 | 0.41 | 5.61 |
|  |  | Moderate activity (HBI 8–16) | 8 | 0 | 0.00 | 0.00 | - | 0.00 | 0.00 | - |
|  |  | Severe activity (HBI >16) | 1 | 0 | 0.00 | 0.00 | - | 0.00 | 0.00 | - |
| VDZ | Age at the initiation of the first advanced therapy | years | 26 | 12 | 0.97 | 0.94 | 1.01 | 0.04 | 0.00 | - |
|  | Sex | Female | 10 | 6 | Reference |  |  | Reference |  |  |
|  |  | Male | 16 | 6 | 0.51 | 0.16 | 1.58 | 0.00 | 0.00 | - |
|  | Corticosteroid use before the first advanced therapy | No | 21 | 9 | Reference |  |  | Reference |  |  |
|  |  | Yes | 5 | 3 | 3.15 | 0.76 | 12.98 | 0.00 | 0.00 | - |
|  | Immunomodulator use before the first advanced therapy | No | 17 | 9 | Reference |  |  | Reference |  |  |
|  |  | Yes | 9 | 3 | 0.43 | 0.12 | 1.62 | 0.00 | 0.00 | - |
|  | Early/delayed initiation of an advanced therapy | Delayed initiation | 21 | 8 | Reference |  |  | Reference |  |  |
|  |  | Early initiation | 5 | 4 | 4.54 | 1.27 | 16.25 | 9.30E74 | 0.00 | - |
|  | Intestinal resection surgery before the first advanced therapy | No | 23 | 11 | Reference |  |  | Reference |  |  |
|  |  | Yes | 3 | 1 | 0.59 | 0.08 | 4.60 | 2.18E28 | 0.00 | - |
|  | Disease location | L2: Colonic | 7 | 3 | Reference |  |  | Reference |  |  |
|  |  | L1: Ileal | 7 | 2 | 0.41 | 0.07 | 2.51 | 9.28E31 | 0.00 | - |
|  |  | L3: Ileocolonic | 11 | 7 | 1.30 | 0.34 | 5.07 | 5.05E32 | 0.00 | - |
|  | Disease behaviour | B1: Non-stricturing, non-penetrating disease | 11 | 9 | Reference |  |  | Reference |  |  |
|  |  | B2: Stricturing disease | 9 | 2 | 0.13 | 0.03 | 0.63 | 0.00 | 0.00 | - |
|  |  | B3: Penetrating disease | 6 | 1 | 0.14 | 0.02 | 1.14 | 0.00 | 0.00 | - |
|  | Perianal lesion | No | 18 | 9 | Reference |  |  | Reference |  |  |
|  |  | Yes | 8 | 3 | 0.75 | 0.20 | 2.78 | 7.35E52 | 0.00 | - |
|  | Haemoglobin concentration | ≥12.5 g/dL | 18 | 9 | Reference |  |  | Reference |  |  |
|  |  | <10 g/dL | 1 | 1 | 21.87 | 1.35 | 354.40 | 0.00 | 0.00 | - |
|  |  | 10–12.49 g/dL | 7 | 2 | 0.60 | 0.13 | 2.77 | 0.00 | 0.00 | - |
|  | Serum albumin concentration | ≥4 g/dL | 16 | 3 | Reference |  |  | Reference |  |  |
|  |  | <3 g/dL | 0 | 0 | - | - | - | - | - | - |
|  |  | 3–3.99 g/dL | 10 | 9 | 15.46 | 3.17 | 75.35 | 1.92E24 | 0.00 | - |
|  | C-reactive protein concentration | 3 mg/L | 26 | 12 | 1.57 | 1.19 | 2.07 | 3.37E10 | 0.00 | - |
|  | Severity | Remission (HBI<5) | 13 | 4 | Reference |  |  | Reference |  |  |
|  |  | Mild activity (HBI 5–7) | 6 | 4 | 3.00 | 0.75 | 12.11 | 0.00 | 0.00 | - |
|  |  | Moderate activity (HBI 8–16) | 1 | 1 | 2032902393550.58 | 0.00 | - | 0.00 | 0.00 | - |
|  |  | Severe activity (HBI >16) | 0 | 0 | - | - | - | - | - | - |
| *1 Hazard ratio for each risk factor from the Cox proportional hazards model.  *2 Hazard ratio for each risk factor from the multivariate Cox proportional hazards model. | | | | | | | | | | |

ADA, adalimumab; CI, confidence interval; HBI, Harvey–Bradshaw index; HR, hazard ratio; IFX, infliximab; IMM, immunomodulators; UST, ustekinumab; VDZ, vedolizumab.

Table S2. Patient characteristics at the initiation of the first advanced therapy by treatment group after propensity score adjustment using inverse probability of treatment weighting

| Total *n* = 1106 |  | At the initiation of the first advanced therapy, *n* = 1106 (100.0%) | | | |
| --- | --- | --- | --- | --- | --- |
|  |  | All advanced therapies | Infliximab | Adalimumab | Ustekinumab |
|  |  | *n* (%) | *n* (%) | *n* (%) | *n* (%) |
| Age at the initiation of the first advanced therapy (years) | *n* | 1106 | 359 | 369 | 377 |
|  | Mean ± SD | 27.8 ± 11.6 | 27.8 ± 10.0 | 28.3 ± 12.7 | 27.4 ± 11.9 |
| Period of time from a diagnosis of CD to the initiation of the first advanced therapy (months) | *n* | 1106 | 359 | 369 | 377 |
|  | Mean ± SD | 6.30 ± 8.71 | 3.81 ± 5.27 | 6.15 ± 8.16 | 8.82 ± 10.91 |
| BMI (kg/m^2^) | *n* | 1106 | 359 | 369 | 377 |
|  | Mean ± SD | 20.58 ± 3.55 | 20.78 ± 3.96 | 20.42 ± 2.96 | 20.56 ± 3.65 |
| Disease location | *n* | 1106 | 359 | 369 | 377 |
|  | L1: Ileal | 292 (26.4%) | 103 (28.6%) | 95 (25.6%) | 95 (25.1%) |
|  | L2: Colonic | 172 (15.5%) | 55 (15.3%) | 58 (15.7%) | 59 (15.5%) |
|  | L3: Ileocolonic | 642 (58.1%) | 202 (56.1%) | 217 (58.7%) | 224 (59.3%) |
| Disease behaviour | *n* | 1106 | 359 | 369 | 377 |
|  | B1: Non-stricturing, non-penetrating | 615 (55.6%) | 201 (55.8%) | 214 (57.9%) | 201 (53.2%) |
|  | B2: Stricturing | 334 (30.2%) | 115 (32.0%) | 104 (28.2%) | 115 (30.5%) |
|  | B3: Penetrating | 157 (14.2%) | 44 (12.2%) | 51 (13.9%) | 62 (16.3%) |
| Perianal lesion | *n* | 1106 | 359 | 369 | 377 |
|  | No | 586 (53.0%) | 193 (53.6%) | 199 (53.8%) | 195 (51.7%) |
|  | Yes | 520 (47.0%) | 167 (46.4%) | 170 (46.2%) | 182 (48.3%) |
| C-reactive protein (mg/L) | *n* | 1106 | 359 | 369 | 377 |
|  | Mean ± SD | 1.8176 ± 2.2585 | 1.9285 ± 2.2964 | 1.8836 ± 2.4382 | 1.6476 ± 2.0232 |
| HBI | *n* | 497 | 158 | 168 | 172 |
|  | Mean ± SD | 5.1 ± 4.1 | 5.0 ± 4.1 | 5.1 ± 3.9 | 5.1 ± 4.1 |
| Disease severity | *n* | 497 | 158 | 168 | 172 |
|  | Remission (HBI<5) | 258 (51.9%) | 85 (53.8%) | 85 (50.4%) | 88 (51.5%) |
|  | Mild activity (HBI 5–7) | 137 (27.5%) | 43 (27.4%) | 48 (28.4%) | 46 (26.8%) |
|  | Moderate activity (HBI 8–16) | 94 (18.9%) | 28 (17.5%) | 31 (18.7%) | 35 (20.4%) |
|  | Severe activity (HBI >16) | 8 (1.7%) | 2 (1.3%) | 4 (2.4%) | 2 (1.3%) |

The inverse probability of treatment weighting method, based on propensity score, was used.

Propensity scores were estimated using a multinomial logistic regression model including sex, the age at the initiation of the first advanced therapy, BMI, CRP, disease location, disease behaviour, the presence of perianal lesions, disease severity (HBI), and prior corticosteroid use.

Standardized differences before and after IPTW adjustment are presented to identify any differences in covariates.

BMI, body mass index; CRP, C-reactive protein concentration.

**Figure S1. Forest plot of the factors associated with persistence with the use of the first-line advanced therapy**

CD, Crohn’s disease; CI, confidence interval; CRP, C-reactive protein concentration; HBI, Harvey–Bradshaw index; 5-ASA, 5-aminosalicylic acid.

Figure S2. Persistence with the first-line advanced therapy, according to the timing of initiation (≤6 months *vs*. >6 months after a diagnosis)

Unadjusted Kaplan–Meier curves using a 6-month threshold are shown, as a sensitivity analysis.
